# Predicting Late Recurrence of Atrial Fibrillation After Radiofrequency Ablation in Patients With Atrial Fibrillation: Comparison of C2HEST and HATCH Scores

**DOI:** 10.3389/fcvm.2022.907817

**Published:** 2022-06-21

**Authors:** Jingjing Han, Guangling Li, Demei Zhang, Xiaomei Wang, Xueya Guo

**Affiliations:** ^1^Department of Cardiology, Lanzhou University Second Hospital, Lanzhou University, Lanzhou, China; ^2^Lanzhou University Second Hospital, The Second Clinical Medical College of Lanzhou University, Lanzhou, China

**Keywords:** atrial fibrillation, radiofrequency ablation, hypertension, heart failure, C2HEST scores, HATCH scores

## Abstract

**Objective:**

This study was aimed to investigate the risk of recurrence in patients with atrial fibrillation (AF) after radiofrequency ablation and predict risk of recurrence using C2HEST and HATCH scores.

**Methods:**

We retrospectively included 322 patients with AF from Second Hospital of Lanzhou University, and 261 patients were included in the analysis finally. They had AF and were admitted for radiofrequency catheter ablation. We compared the ability of C2HEST and HATCH scores to predict recurrence after radiofrequency ablation of AF. The predictive ability of C2HEST and HATCH scores for AF recurrence was estimated by the area under the receiver operating characteristic curve (AUROC). The difference in receiver operating characteristic curve between the two models was compared using the DeLong test.

**Results:**

Of the 261 patients included in the analysis, 83 (31.6%) patients suffered a late recurrence of AF after radiofrequency ablation. The risk of postoperative recurrence of AF increased with increasing C2HEST and HATCH scores. The AUROC of C2HEST and HATCH scores in predicting postoperative recurrence of AF was 0.773 (95%CI, 0.713–0.833) and 0.801 (95% CI, 0.740–0.861), respectively. There was no significant difference between the two models in their ability to evaluate patients for postoperative recurrence of AF (DeLong test *p*-value = 0.36). Among the risk factors in both models, hypertension and heart failure (HF) contributed the most to postoperative recurrence after AF, and higher blood pressure and lower cardiac ejection fraction (EF) were associated with a higher risk of recurrence.

**Conclusion:**

Both C2HEST and HATCH scores were significantly associated with the risk of late recurrence after radiofrequency ablation of AF. Besides hypertension and HF contributed the most to postoperative recurrence after AF.

## Introduction

Atrial fibrillation (AF) is one of the most common clinical arrhythmias. Epidemiological studies show that about 2% of the world’s population suffers from AF (1). It is estimated that by 2050, there will be 6–12 million patients with AF in the United States, and by 2060, there will be 17.9 million patients with AF in Europe (2). AF can cause heart failure (HF), ischemic stroke (IS), and dementia, increase morbidity and mortality in this population, and cause a significant disease burden (3). In existing studies, several models for predicting new-onset AF have been validated. However, most of them are based on Western demographic data and have not been further confirmed in Asian populations (4–8). More importantly, to the best of our knowledge, these models are rarely or not used to predict the recurrence of AF after radiofrequency ablation. Radiofrequency ablation of AF, as one of the most effective ways to control AF rhythm and maintain sinus rhythm, has been recommended as a first-line treatment in many guidelines (9, 10). However, due to insufficient evidence, the existing AF guidelines do not recommend using these models to provide a reference for radiofrequency catheter ablation of AF. In addition, current studies have shown that the occurrence and maintenance of AF depend on electrophysiological substrates (11). Therefore, we can put forward a reasonable hypothesis in which models used to predict the occurrence of AF can also predict the recurrence of AF after radiofrequency ablation, which is very important to guide the individualized treatment of AF patients.

The C2HEST score is used to predict the risk of developing AF in people without structural heart disease: C2: coronary artery disease (CAD) and chronic obstructive pulmonary disease (COPD) (each gets 1 point), H: hypertension (1 point), E: elderly (age ≥ 75 years, 2 points), S: systolic HF (2 points), and T: thyroid disease (hyperthyroidism, 1 point). The HATCH score is applied to predict the risk of progression to persistent AF in patients with paroxysmal AF: H: hypertension (1 point), A: age > 75 years (1 point), T: transient ischemic attack (TIA) or cerebrovascular accident (CVA) (2 points), C: COPD (1 point), H: heart failure (2 points) (12–17).

The predictive value of C2HEST and HATCH models for new-onset AF has been demonstrated in Asian populations. In many studies, the value of the C2HEST score in predicting the occurrence of AF is superior to other prediction models (16, 18). However, as far as we know, there is no study using the C2HEST scoring system to predict the risk of late recurrence after radiofrequency ablation of AF, and the evidence using the HATCH score to predict recurrence after radiofrequency ablation in patients with AF is also very limited. For patients with AF, predicting the risk of recurrence after radiofrequency ablation is very important for selecting individualized treatment for patients. However, understanding which patients will benefit from ablation remains a considerable challenge. Therefore, the C2HEST and HATCH scores were used to predict the recurrence risk after radiofrequency ablation in patients with AF, and their predictive power was compared.

## Materials and Methods

### Data Sources and Trial Design

The study was a retrospective cohort study with strict inclusion and exclusion criteria. It was approved by the Ethics Committee of the Second Hospital of Lanzhou University with visa-free informed consent. All patients have signed informed consent for radiofrequency ablation. In the Cardiovascular Department of the hospital, we collected patients who underwent radiofrequency ablation for AF from April 2017 to July 2020. The privacy of all subjects was fully protected, and the patient’s private information was encrypted before this data was released. The study adheres to the principles outlined in the Declaration of Helsinki.

### Patient Selection and Follow-Up

We retrospectively recruited consecutive patients with AF in the electronic medical record database of the Second Hospital of Lanzhou University. Based on the purpose of our study, the inclusion criteria for subjects were as follows: 1. The patient’s age is greater than 18 years old. 2. The patient first presented with AF on the 24-h ambulatory electrocardiogram or standard 12-lead electrocardiogram (ECG) during this admission, and the diagnosis was based on the International Classification of Diseases, Ninth Revision, Clinical Modification (ICD-9-CM). 3. The patient meets the indications for radiofrequency ablation of AF. Subject exclusion criteria: (1) The patient has a history of valvular heart disease (e.g., valve replacement, valvular disease related to hemodynamics). (2) The patient has undergone previous operations for AF (such as radiofrequency ablation, cryo-balloon, and surgical maze procedures). (3) The patient’s preoperative transesophageal echocardiography showed a left atrial thrombus. (4) The patient is in the active stage of hemorrhagic disease, systemic infection, or organ failure and cannot tolerate the operation. (5) Radiofrequency ablation was rejected by patients with AF. Primary indications for radiofrequency ablation in patients with AF: (1) The patient has frequent episodes of symptoms, and at least one class I or class III antiarrhythmic drug treatment is ineffective or intolerable. (2) Patients with frequent symptoms and unwilling to take drug treatment. (3) The patient is asymptomatic, but after comprehensively considering the efficacy and risk of drug and catheter ablation, catheter ablation is more beneficial. For all included subjects, 3 months after radiofrequency ablation of AF was used as a blank period and did not participate in follow-up. Within 3 months after the operation, all patients had been prescribed amiodarone for 3 months according to the guidelines recommended. Follow-up was started 3 months after radiofrequency ablation, and the minimum follow-up was 3 times (3 months, 6 months, and 1 year after operation). When the patient did not follow up on time, the patient was contacted by phone and advised to follow up on time. In addition, when the patient has symptoms, he will be admitted to the hospital for ECG examination immediately or outpatient ECG examination at any time when the patient needs it. ECG diagnosis and follow-up time were recorded during the examination. The most prolonged follow-up period for patients is 9 months, and the follow-up is stopped after the endpoint event occurs, and the follow-up time ended in July 2021. AF was diagnosed according to the International Classification of Diseases, Ninth Revision, Clinical Modification (ICD-9-CM). C2HEST and HATCH scores were calculated for each subject to predict the risk of AF recurrence. For each risk factor in the C2HEST and HATCH scores, the International Classification of Diseases, Ninth Revision, Clinical Modification (ICD-9-CM) was used as the diagnostic criteria.

### Ablation Procedure

The main radiofrequency ablation procedure was circumferential pulmonary vein isolation. A three-dimensional mapping system was used in all patients (NavX or CARTO). The endpoint of the operation was a bidirectional block of pulmonary vein and left atrium, which was recorded by mapping or ablation catheter or verified by pacing. Radiofrequency (RF) pulses were delivered using a 3.5 mm cooled-tip catheter, with a temperature setting up to 45 degree centigrade and an energy up to 42 W. When ablation was performed in the posterior wall, RF power was reduced to 25 W to reduce the risk of injuring the surrounding structures. Additional substrate modification (linear ablations or complex fractionated electrogram-guided ablations) was left to the discretion of the operating electrophysiologist. A fairly conservative strategy for additional ablations was followed. Cavotricuspid isthmus block was performed in patients with documented typical atrial flutter.

### Outcomes

The study was a single outcome. The endpoint was the patient’s late recurrence of AF after radiofrequency ablation during the 9-month follow-up. Late postoperative recurrence is defined as rapid atrial arrhythmias such as AF, atrial tachycardia, or atrial flutter found on ECG or ambulatory ECG within 3–12 months after radiofrequency ablation of AF, with a duration of more than 30 s (9).

### Statistical Analysis

In the baseline characteristics of the included subjects, categorical variables are represented by absolute numbers (percentages), continuous variables that satisfy the normal distribution are represented by the mean (standard deviation), and those that do not satisfy the normal distribution are represented by the median (Interquartile range). Normality was assessed using the Kolmogorov-Smirnov method, QQ plot and histogram. The recurrence rate of patients with AF after radiofrequency ablation was calculated by multiplying the number of events of atrial fibrillation recurrence by the survival time divided by the total number of follow-up person-months. Both C2HEST and HATCH scores were divided into three groups: 0, 1, and greater than or equal to 2. The Kaplan-Meier curves of C2HEST and HATCH scores under different scores were drawn, and the ordinate was the cumulative risk of postoperative recurrence of AF. Furthermore, we use the Logrank test to detect whether the curves under different scores are different. The hazard ratio (HR) and 95% CI of C2HEST and HATCH scores for predicting postoperative recurrence of AF under different scores were calculated by Cox regression, the model effect was evaluated by −2 log-likelihood, and the overall model was tested by Omnibus test. Then, other risk factors were included in different groups, the model was adjusted, and adjusted HR and their 95% CI were calculated. The area under the receiver operating characteristic curve was used to estimate the predictive power of the C2HEST and HATCH models for AF recurrence, and the Hosmer-Lemeshow goodness-of-fit test was used to evaluate the model. The DeLong test was used to compare the area under the receiver operating characteristic curve between two different prediction models. Statistical analysis was performed using SPSS 24.0 or R software. The drawing of pictures was realized using SPSS 24.0 version or R software. The significance level of the two-sided test for the *p*-value was set at 0.05.

## Results

### Trial Population

**FIGURE 1:**
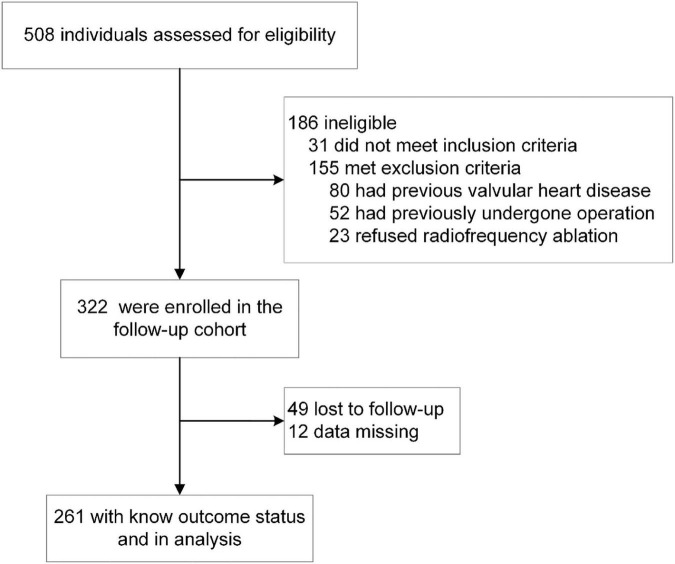
Screening and follow-up.

From April 2017 to July 2020, a total of 322 patients with a first-time diagnosis of AF were enrolled at the Second Hospital of Lanzhou University (Figure 1). All patients underwent radiofrequency catheter ablation of AF. 49 patients were lost to follow-up and data were missing for 12 patients. A total of 261 patients were finally included in the analysis.

Among the included patients, the mean age was 58.4 years, and 36.0% were female. Among them, there were 12 patients (12.6%) with COPD, 51 patients (19.5%) with CAD, 118 patients (45.2%) with hypertension, 71 patients with HF (27.1%), 5 patients with hyperthyroidism (1.9%) and 23 patients (8.8%) with CVA or TIA. The patient’s C2HEST score was 1 (IQR, 0–1), and the HATCH score was 1 (IQR, 0–2). The median follow-up of patients was 9 months (Table 1).

**TABLE 1:** Baseline characteristics of study subjects.

Variables	*N* = 261
**Age, years**	
≤64	189 (72.4%)
65–74	66 (25.3%)
≥75	6 (2.3%)
Mean ± *SD*	58.44 ± 9.44
**Gender**	
Male	167 (64.0%)
Female	94 (36.0%)
Type of AF	
Paroxysmal AF	108
Persistent AF	153
**Risk factors (Components of C2HEST and HATCH scores)**	
COPD	12 (4.6%)
CAD	51 (19.5%)
Hypertension	118 (45.2%)
Hypertension grade 1	16 (6.1%)
Hypertension grade 2	32 (12.3%)
Hypertension grade 3	70 (26.8%)
HF	71 (27.1%)
HFrEF	4 (1.5%)
HFmrEF	9 (3.4%)
HFpEF	58 (22.2%)
Hyperthyroidism	5 (1.9%)
TIA or CVA	23 (8.8%)
**Other risk factors**	
Hyperlipidemia	43 (16.5%)
Hyperuricemia	18 (6.9%)
Diabetes	56 (21.5%)
OSAHS	12 (4.6%)
CKD	8 (3.1%)
**Echocardiography, mm (preoperative)**	
LA	40.3 (36.0–46.3)
RA	39.0 (35.1–45.0)
LVEF	60.0 (55.5–65.0)
PFO	32 (12.3%)
E/E’	10.6 (10.0–11.2)
C2HEST score	1 (0–1)
HATCH score	1 (0–2)
CHA2DS2-VASc score	2 (1–3)
Follow-up for AF recurrence, months	9 (6–9)

*Values are presented as n (%), mean (SD), or median (IQR). COPD, chronic obstructive pulmonary disease; CAD, coronary artery disease; HFrEF, heart failure with reduced ejection fraction; HFmrEF, heart failure with mid-range ejection fraction; HFpEF, heart failure with preserved ejection fraction; TIA, transient ischemic attack; CVA, cerebrovascular accident; OSAHS, obstructive sleep apnea hypopnea syndrome; CKD, chronic kidney disease; LVEF, left ventricular ejection fraction. PFO, patent foramen ovale.*

### Outcomes

In the Cox regression proportional hazards model, patients with C2HEST scores of 0, 1, and ≥ 2 had AF postoperative recurrence rate of 3.01/Per 100 person-months, 9.10/Per 100 person-months, and 6.03/Per 100 person-months, respectively. Before adjusting for confounders, patients with a C2HEST score of 0, 1, and ≥ 2 had Crude HR of 1.00 (reference), 5.30 (95%CI, 2.56–10.98), and 12.69 (95%CI, 6.09–26.4), respectively. After adjusting for relevant confounding factors, the adjusted HR was 1.00 (reference), 4.41 (95% CI, 2.10–9.26), and 12.72 (95% CI, 6.00–27.00) (Table 2).

**TABLE 2:** Incidence and hazard ratios of atrial fibrillation late recurrence stratified by C2HEST score.

C2HEST score	*N*	No. of events	Person-months^&^	Rate^#^	Crude HR	95%CI	Adjusted HR*	95%CI
0	106	9	59	3.01	1.00	Reference	1.00	Reference
1	99	38	178	9.10	5.30	2.56–10.98	4.41	2.10–9.26
≥2	56	36	118	6.03	12.69	6.09–26.40	12.72	6.00–27.00
P for trend					*P* < 0.001		*P* < 0.001	

*^#^Per 100 person-months.*

*Adjusted for gender, TIA or CVA, hyperlipidemia, hyperuricemia, OSAHS, CKD, LA, RA, PFO.

*^&^Person-months of recurrent events after atrial fibrillation radiofrequency ablation.*

Similarly, patients with HATCH scores of 0, 1, and ≥ 2 had AF postoperative recurrence rates of 3.32/Per 100 person-months, 4.24/Per 100 person-months, and 10.60/Per 100 person-months, respectively. Before adjusting for confounders, Crude HR was 1.00 (reference), 2.65 (95%CI, 1.20–5.84), and 8.58 (95%CI, 4.37–16.83) for patients with a HATCH score of 0, 1, and ≥ 2, respectively. After adjusting for confounding factors, adjusted HR was 1.00 (reference), 2.20 (95% CI, 2.10–9.26) and 7.44 (95%CI, 3.70–14.94) (Table 3).

**TABLE 3:** Incidence and hazard ratios of atrial fibrillation late recurrence stratified by HATCH score.

HATCH score	*N*	No. of events	Person-months^&^	Rate^#^	Crude HR	95%CI	Adjusted HR*	95%CI
0	99	10	65	3.32	1.00	Reference	1.00	Reference
1	64	16	83	4.24	2.65	1.20–5.84	2.20	0.98–4.93
≥2	98	57	207	10.60	8.58	4.37–16.83	7.44	3.70–14.94
P for trend					*P* < 0.001		*P* < 0.001	

*^#^Per 100 person-months.*

*Adjusted for gender, CAD, hyperlipidemia, hyperuricemia, OSAHS, CKD, LA, RA, PFO.

*^&^Person-months of recurrent events after atrial fibrillation radiofrequency ablation.*

**FIGURE 2:**
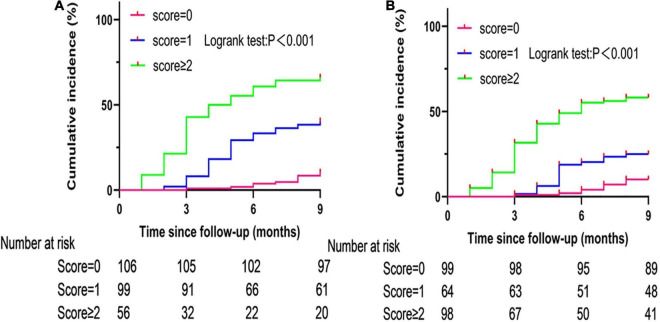
Cumulative incidence curves of atrial fibrillation recurrence stratified by C2HEST **(A)** and HATCH **(B)** scores.

As seen in the Kaplan Meier curve, the cumulative risk of postoperative recurrence of AF increased with the extension of follow-up time under different scores of the C2HEST and HATCH models. Figure 2 show that the cumulative risk of postoperative recurrence of AF presents different trends under different scores. Patients with C2HEST scores of 0, 1, and ≥ 2 had a gradient increase in the cumulative risk of postoperative recurrence of AF, with a Logrank test *p*-value < 0.001.

### Evaluation of Predictive Models and Cox Regression Analysis of Risk Factors

**FIGURE 3:**
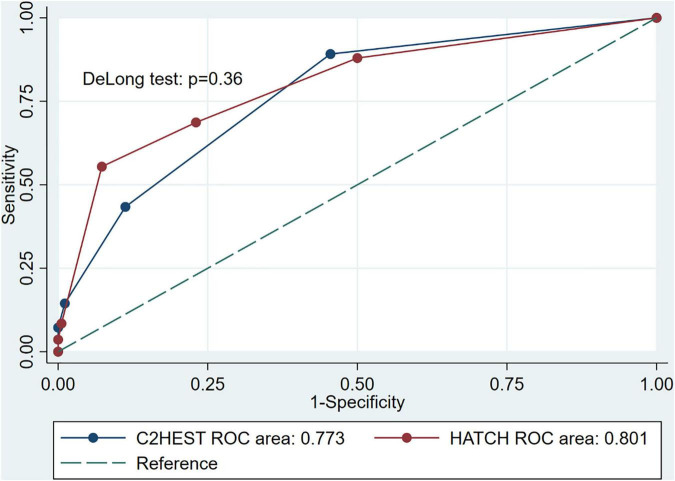
Receiver operating characteristic curves for the C2HEST and HATCH score in predicting incident atrial fibrillation recurrence, and the DeLong test was used to compare the predictive power between C2HEST and HATCH score.

It can be seen from the receiver operating characteristic curve that both C2HEST and HATCH scores can effectively predict the recurrence of AF, and the areas under the curve are 0.773 (95%CI, 0.713–0.833) and 0.801(95% CI, 0.740–0.861), respectively (Figure 3). Using the DeLong test with a *p*-value of 0.36, there was no statistical difference in the ability of the two prediction models to predict postoperative recurrence of AF.

**FIGURE 4:**
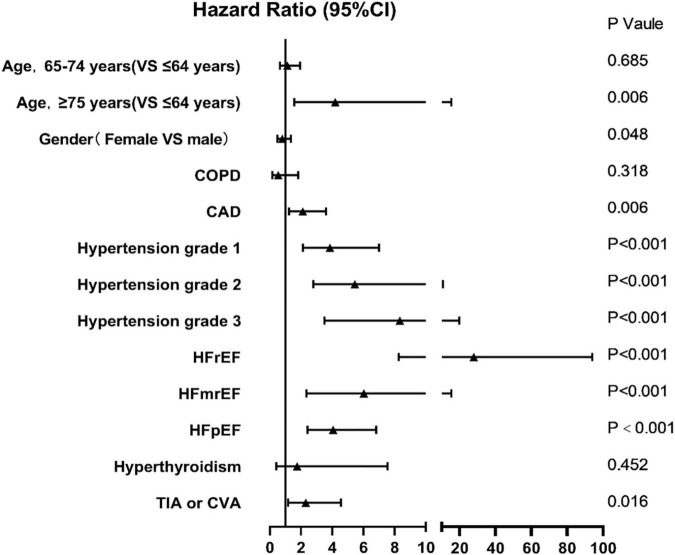
Hazard Ratio for the outcome and its risk factors in C2HEST and HATCH scores.

In the forest plot displayed by Cox regression, we can see that the difference in gender and age (65–74 years) did not lead to an increase in the recurrence rate of AF, with HR of 0.80 (95%CI, 0.48–1.35, *P* = 0.05), 1.12 (95%CI, 0.65–1.94, *P* = 0.69), respectively (Figure 4). COPD and hyperthyroidism also did not increase the risk of AF recurrence, with HR of 0.54 (0.16–1.82, *P* = 0.32) and 1.75 (0.41–7.54, *P* = 0.45), respectively. Moreover, other risk factors in C2HEST and HATCH scores can increase the risk of postoperative recurrence of AF. Among them, hypertension and heart failure caused the highest risk of postoperative recurrence of AF and showed a trend that the higher the blood pressure and the lower ejection fraction, the higher the risk of postoperative recurrence of AF.

## Discussion

This study is the first to evaluate the risk of recurrence after radiofrequency ablation of AF using the C2HEST score. It provided evidence that the score can be used as a predictor of the risk of postoperative recurrence of AF. In the model, the area under the receiver operating characteristic curve is 0.733 (95% CI, 0.713–0.833), indicating that the model is a good predictor. At the same time, we should note that in this model, the risk of AF recurrence increased with increasing C2HEST score (Logrank test *p* < 0.001). Similar findings were also found in the HATCH score, and there was no statistical difference in the ability of the two models to predict postoperative recurrence of AF (DeLong test *p* = 0.36). We note in the C2HEST and HATCH scores. The most significant differences between the two scores are TIA, CVA, CAD, and Hyperthyroidism, while the rest of the risk factors are the same. CAD and Hyperthyroidism were added to the C2HEST score, while TIA and CVA were added to the HATCH score. So the similarity in predictive power between the two is explicable. However, some controversies in the HATCH score must be pointed out. As far as we know, three studies have examined the relationship between HATCH score and recurrence after catheter ablation of AF, of which there is no multicenter study with large sample size. In the study of Tang et al. the HATCH score was considered to have no predictive value for postoperative recurrence of AF (19). Through comparison, we found the possible reasons for the difference in results. The subjects in this study were operated on from January 2005 to September 2007. Since then, radiofrequency ablation of AF has been dramatically developed, while this may have an impact on the findings of the study. In addition, we found that patients were not strictly included and excluded in this study, and the duration of AF, the type of AF, the screening method of AF follow-up, and whether they were complicated with valvular heart disease all affect the risk of postoperative recurrence (20, 21). More importantly, the study’s operative endpoint required only an entry block when defining pulmonary vein isolation, whereas our operative endpoint required both an entry and exit block. Bidirectional blockade allows complete pulmonary vein isolation and longer pulmonary vein isolation, which directly affects the trial’s outcome (22–24). In the study of Mulder et al. it was also concluded that the HATCH score had no predictive value for the recurrence of AF (25). In this article, we found that it did not exclude patients with previous atrial fibrillation (patients may be persistent atrial fibrillation or long-standing persistent atrial fibrillation) or patients with valvular heart disease, which may increase the recurrence rate and reduce the prediction ability of the model. Secondly, the main inclusion criteria is that patients should have the results of cardiac CT or cardiac MRI before radiofrequency ablation, and patients with poor image quality are excluded, which may lead to selection bias. In terms of follow-up time, the median time of patients was 29 (IQR, 12–68) months. However, the longer the time of AF, the greater the possibility of postoperative recurrence of AF, which will further reduce the prediction ability of the model (26). Different from the above study, in the study of Bai et al. we reached a similar conclusion that the HATCH score can predict the risk of postoperative recurrence of AF (27). In addition, consistent with our results, they also believe that among the risk factors included in the model, HF and hypertension make a significant contribution to the postoperative recurrence of AF. However, we note that the area under the receiver operating characteristic curve of this study is 0.58 (95% CI, 0.52–0.63), which is relatively poor in predictive power compared to our findings. After further comparison, it was found that the study population was patients with AF combined with pulmonary disease, and there was no previous research data in this area. The extent to which it will affect the results of the study needs further research. At the same time, the study did not mention the exclusion criteria of patients, which would lead to a further increase in the variability of the results and a further decrease in the predictive power. In addition, the median follow-up time of the study was 6 months after the operation, which seems to be insufficient for the evaluation of late recurrence of AF. More importantly, the endpoint of the study was the duration of AF > 30 s, which did not seem to include other atrial tachyarrhythmias, which would significantly impact the study’s outcome. Unlike the above three studies, we note that the age of the included population in our existing study is relatively small (58.44 ± 9.44 years), which may also lead to the decline of confounding factors for the study outcome, improving the predictability of the model.

In our study, patients aged 65–74 years did not have an increased risk of recurrence after radiofrequency ablation of AF compared with age ≤ 64 years. Patients ≥ 75 years of age are at increased risk of recurrence. In a recent study by Li et al. it was found that the modified mC2HEST score (with age ≥ 65 years added as 1 point) increased the predictive power for atrial fibrillation (28). However, from our results, this was not observed for postoperative recurrence of AF. In addition, elderly patients with AF have a reduced ability to clear and metabolize antiarrhythmic drugs and are prone to proarrhythmic effects and drug-related bradycardia. Therefore, radiofrequency catheter ablation of AF can be considered in the elderly (29). At the same time, several research results suggest that the success rate of catheter ablation in patients aged ≥ 75 years is similar to that in young patients, while there is no difference in the incidence of complications. However, our study suggests that for patients aged ≥ 75 years, the risk of late postoperative recurrence of AF is increased, which provides another consideration for clinicians’ decision-making. Of course, this needs to be further verified in a large sample and multicenter studies.

In our study, hypertension also leads to increased recurrence of AF after radiofrequency ablation, which has also been confirmed in other studies (30). However, there is currently insufficient evidence that blood pressure control improves the success rate of catheter ablation of AF. In recent years, catheter ablation has achieved an apparent curative effect in patients with AF complicated with HF, and its success rate is similar to that in patients with AF without HF. At the same time, in the sinus rhythm maintenance group, the postoperative cardiac function indexes and quality of life were significantly improved, while the incidence of perioperative complications had no significant difference compared with those without HF (31–33). In addition, the CASTLE-AF study showed that for patients with HF complicated with AF, the composite endpoint of all-cause death or hospitalization due to deterioration of HF in patients with catheter ablation was significantly lower than that of drug treatment (31). However, it should be noted that patients with HF who had AF have a higher recurrence rate and complication rate after catheter ablation due to cardiac remodeling and combined structural heart disease. This was also confirmed in our study, where HF contributed the most to the late recurrence of AF in both models, and the lower the ejection fraction, the higher risk of recurrence. However, the sample size of our study was small, especially for patients with HFrEF, so further large-sample studies are necessary. In addition, for some special cases, it should be pointed out that for AF caused by worsening HF, a rhythm control strategy is not superior to ventricular rate control, and catheter ablation is an optional strategy (34). At this point, two predictive models can help clinicians make further decisions. For CAD, TIA or CVA can promote the recurrence of AF, which has also been verified in previous studies (16, 35). In our study, they promoted postoperative recurrence of AF, but their contribution was limited.

The clinical significance of this study is mainly that C2HEST and HATCH scores can guide the decision-making process of clinicians. First, patients with high C2HEST and HATCH scores are at increased risk of recurrence, requiring more frequent monitoring. In addition, patients with high C2HEST and HATCH scores tend to choose procedures with lower recurrence rates. Conversely, clinicians largely have to consider the risk of recurrence and the economic cost-effectiveness of patients after catheter ablation. Therefore, excessive radiofrequency ablation may be avoided in patients with high C2HEST and HATCH scores. Obviously, the clinical value of the C2HEST and HATCH scores needs further clinical trial validation.

### Study Limitations

First, the study was a single-center study, and a retrospective cohort study was used in the trial design. Therefore, the study may not be extrapolated to other centers. At the same time, the study used radiofrequency catheter ablation for AF, so it may not be extrapolated to other operation treatments. However, this does not deny the original clinical significance of the study. Second, the study had a short follow-up period and did not perform continuous regular monitoring of postoperative AF recurrence. Several studies have shown that many patients with postoperative recurrence of AF are asymptomatic, which may lead to underestimating the risk of postoperative recurrence in patients and affect the evaluation of the model (36–38). Third, while helping to clarify the nature of the problem, strict inclusion and exclusion criteria limit the extrapolation of conclusions across different AF patients. Fourth, although we screened 508 samples and included 322 patients with AF when we performed the analysis, especially the sample size of patients aged ≥ 75 years and HFrEF was small, resulting in a wide CI for the HR, which has a certain impact on the accuracy of the trial conclusion and requires further validation by a large sample study. However, in our opinion, this does not reduce the overall predictive power of the C2HEST and HATCH scores.

## Conclusion

Both C2HEST and HATCH scores are reliable predictors of recurrence after radiofrequency ablation of AF, and there is no difference in predictive ability between the two models. Besides, we found that hypertension and HF contributed the most among the risk factors in both models. C2HEST and HATCH scores can provide a reference for clinicians’ decision-making process.

## Data Availability Statement

The raw data supporting the conclusions of this article will be made available by the authors, without undue reservation.

## Ethics Statement

The studies involving human participants were reviewed and approved by the Ethics Review Committee of the Second Hospital of Lanzhou University. The ethics committee waived the requirement of written informed consent for participation. Written informed consent was not obtained from the individual(s) for the publication of any potentially identifiable images or data included in this article.

## Author Contributions

JH completed the follow-up of patients and recorded the basic data of patients. GL completed the data analysis and wrote the manuscript. XG provided guidance in this clinical trial. All authors listed have made a substantial, direct, and intellectual contribution to the work, and approved it for publication.

## Conflict of Interest

The authors declare that the research was conducted in the absence of any commercial or financial relationships that could be construed as a potential conflict of interest.

## Publisher’s Note

All claims expressed in this article are solely those of the authors and do not necessarily represent those of their affiliated organizations, or those of the publisher, the editors and the reviewers. Any product that may be evaluated in this article, or claim that may be made by its manufacturer, is not guaranteed or endorsed by the publisher.
